# Strengthening primary health care teams with palliative care leaders: protocol for a cluster randomized clinical trial

**DOI:** 10.1186/s12904-017-0217-9

**Published:** 2017-07-10

**Authors:** Joan Llobera, Noemí Sansó, Amador Ruiz, Merce Llagostera, Estefania Serratusell, Carlos Serrano, María Luisa Martín Roselló, Enric Benito, Eusebio J. Castaño, Alfonso Leiva

**Affiliations:** 1 Primary Care Research Unit of Mallorca, Baleares Health Services-IbSalut, 07005 Palma, Spain; 2 Instituto de Investigación Sanitaria de Palma (IdISPa), 07010 Palma, Spain; 3 Department of Nursing and Physiotherapy, University of Balearic Islands, Valldemossa road 7,5 Km, 07122 Palma, Spain; 4 Sta Catalina Health care centre, Baleares Health Services-IbSalut, Camí de son Cladera 6, 07009 Palma, Spain; 5 Equipo de Soporte a la Atención Domiciliaria Mallorca (ESAD-Mallorca), Baleares Health Services-IbSalut, UBS Es Molinar, c/ Guayaquil 9, Palma, Spain; 6 Baleares Health Services-IbSalut, C/ Jesús 40, 07010 Palma, Spain; 7 Cudeca Hospice Foundation, Av. del Cosmos, S/N, 29631 Malaga, Spain; 8 Balearic Islands Palliative Care Regional Program, Baleares Health Services-IbSalut, C/ Jesús 40, 07010 Palma, Spain; 9 Service of Health Planning of the Health counseling of the Government of the Balearic Islands, Plaza España 9, 07002 Palma de Mallorca, Islas Baleares Spain

**Keywords:** Palliative care, End of life, Integrated care, Public health care, Program development, Outcome assessment, Primary care

## Abstract

**Background:**

The objective of the Balearic Islands Palliative Care (PC) Program is to improve the quality of PC through a shared model consisting of primary health care professionals, home-based PC teams, and PC units in hospitals. According to the World Health Organization (WHO), patients with advanced cancer and other terminal diseases benefit from early identification and proactive PC. We will evaluate the effectiveness of an intervention in which a PC leader is established in the primary health care center, and assess the effect of this intervention on the early identification of patients in need of PC, the efficient use of health care services, and direct health care costs.

**Methods:**

Design: A two-arm cluster randomized clinical trial of 30 Primary Health Care Centers (PHCC) in Mallorca (Spain), in which each center was randomized to an intervention arm or a usual care arm. We expect that the number of patients identified as suitable for PC (including non-oncological PC) is at least 5% greater in the intervention arm.

Sample size: A total of 4640 deceased patients. Outcomes will be assessed by a blinded external review of the electronic records.

Interventions: General practitioners (GPs) and nurse leaders in PC for each PHCC will be appointed. These leaders will help promote PC training of colleagues, improve symptom management and psychological support of patients, and evaluate the complexity of individual cases so that these cases receive assistance from PC home-based teams.

Measurements: Early identification (>90 days before death), evaluation of case complexity, level of case complexity (with referral to a home-based PC team), use and cost of hospital and primary care services, and quality of life during the last month of life (≥2 emergency room visits, ≥2 hospital admissions, ≥14 days of hospitalization).

**Discusion:**

PC leaders in primary care teams will improve the early identification of patients eligible for PC. This initiative could improve the quality of end-of-life care and utilization of hospital resources.

**Trial registration:**

ISRCTN Registry identifier: ISRCTN92479122. Retrospectively registered on 28 February 2017.

## Background

The populations of European countries are getting older, and this is leading to major increases in the number of patients needing palliative care (PC) because of chronic incurable diseases or advanced stages of terminal diseases [1]. The 2013 European Atlas of Palliative Care (EAPC) reported that the use of PC in Europe has increased, although there are variations among countries [2]. Spain, like other European countries, also has an aging population, and thus has initiated an ambitious strategy for PC [3]. There has been wide-spread development of multidisciplinary PC teams in hospitals and primary health care facilities in Spain, and there are currently 284 specific resources for PC, 8 of them in the Balearic Islands [4].

PC was initially proposed for cancer patients [5], but was later extended, although insufficiently, to patients in the later stages of other terminal diseases who had similar needs [6, 7]. Rosenwax [8, 9] classified 9 major causes of death, and this should be considered a minimum required to calculate the needs for PC. Some previous studies explain the development of different illnesses and the care needed during each stage [10–12]. There are models of 3 general groups of patients with chronic progressive illnesses: cancer, organ failure, and dementia/fragility in the elderly. The cancer model is characterized by a short and evident decline, following a variable period of illness with a reasonably predictable decline in physical health. Although cancer can last for many years, the loss of functionality usually only lasts for several months. The organ failure model is characterized by a long period of functional limitations, which can last for about two to five years, in which acute and aggravating episodes occur. Patients usually recover from these acute episodes, although there is an increased loss of functionality. When patients die following an episode, death is typically sudden. The third model of dementia/fragility in the elderly is characterized by a long period of functional decline, with a phase of very low physical and cognitive function that can last for several years. In this model, the decline is progressive and irreversible. Death occurs when the patient is completely dependent on intensive medical care. There are difficulties in identifying the need for PC in these different situations due to differences in the natural courses of the illnesses and the rate of functional status decline.

Initially, caregivers only proposed PC when active treatment could not be prolonged, although it is has long been established that PC must start when active treatment is on-going [13]. There is a general agreement that patients suitable for PC should be identified as soon as possible. The World Health Organization (WHO) emphasized early identification in its definition of PC since 2002: “an approach that improves the quality of life of patients and their families facing the problems associated with life-threatening illness, through the prevention and relief of suffering by means of *early identification* and impeccable assessment and treatment of pain and other problems, physical, psychosocial and spiritual” [14]. Subsequent studies reported that early PC can even prolong survival in patients with some tumors [15, 16]. Thus, the early identification of patients eligible for PC has many benefits, in that aggressive diagnostic and therapeutic interventions are avoided, and unnecessary suffering and costs are reduced [17, 18].

It is important to determine how often PC is needed and the service requirements to calculate the necessary resources. Up to 75% of patient deaths in Spain are due to chronic illnesses from advanced identifiable processes [7]. Murtagh et al. [10] estimated that 63% of deaths were eligible for PC. It is also necessary to determine the incidences of advanced stages of the different pathologies, and this can be estimated from mortality rates. It is also essential to establish the suitable timing for administration of PC [19]. This timing is difficult to predict [20], but an approximation will allow calculation of the prevalence of the problem, planning of services, and estimation of program coverage [21].

The prevalence of cases needing PC will increase with the incidence of the chronic illnesses, the number of eligible pathologies, and the development of PC services. Thus, the need for PC will continue to grow over time. It is therefore essential to identify patients who can only be cared for by conventional services in a primary care or hospital setting, and patients who need specific PC resources (home- or hospital-based), exclusively or in combination with conventional services. The sustainability of the PC model must be based on a shared care model, in which there are general or conventional care services and specialized PC resources [22].

Previous studies assessed the effectiveness of home-based PC in cancer patients [23], but there are fewer studies of PC for patients with chronic but non-oncologic diseases [24]. In Spain, at least at the primary care level, the Spanish Society of Family and Community Medicine and the Spanish Society of Palliative Care reached a consensus regarding their roles in home-based care, and have a long-established commitment to PC [25].

A complex case needing PC [26, 27] is a case with advanced-stage cancer or a terminal illness that is difficult to manage, so that special interventions are needed. Early and accurate identification of patients who require PC would help to identify the need for different PC resources, so that the best response can be provided according to the disease progression and the changing needs of patients. On the other hand, at least in patients with advanced cancer who have a mean survival time of 99 days, three-quarters of this period is spent at home, making the role of the primary care doctor and nurse essential. There are multidisciplinary PC home-based teams, but they should only be required when the complexity of care requires their intervention [21].

In Spain, primary health care doctors, nurses, and other support staff (psychologists, social workers, and others) work in primary health care centers, which care for a mean of 20,000 patient registered. Each doctor and nurse has a mean of 1700 patients registered. The general practitioners (GPs) and community nurses have diverse responsibilities, and must acquire and maintain multiple professional skills so they can appropriately care for patients with diverse health problems. Although each professional cares for assigned patients, there are shared responsibilities within a team, determined by the skills of the health care provider and the medical problem of the patient. PC is a field in which the presence of a leader or expert in each team is considered beneficial, as proposed in the Andalusian and Ballearic Palliative Care Plan [27, 28].

The aim of this randomized clinical trial (RCT) is to assess the effect of the appointment of a PC leader or expert to each health care team who can provide early identification of patients needing PC, encourage the appropriate use of health care services according to the complexity of the case, better satisfy the needs of patients with terminal illnesses, and reduce direct health care costs.

## Methods

### Design

**Fig. 1:**
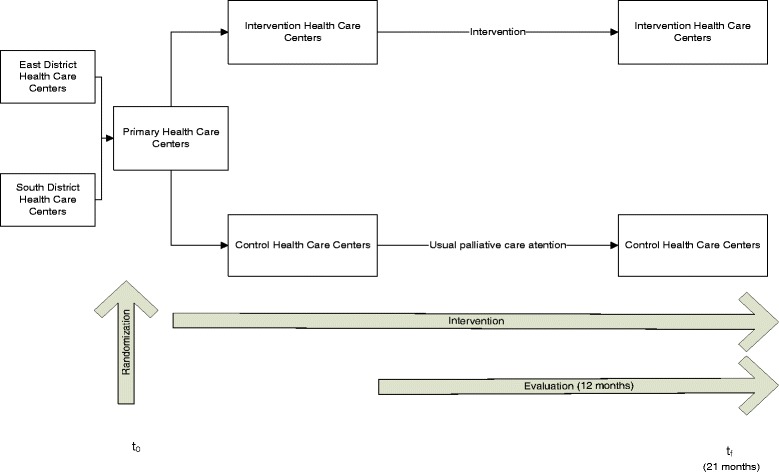
Study design

The study is a multicenter two-armed cluster randomized clinical trial in the primary health care setting. The intervention will be evaluated by external review of the records of deceased patients at 18 months after study onset (Fig. 1).

### Randomization

Each of the 30 participating primary health care centers will be randomly assigned to the intervention or control group. Randomization and concealment will be centralized, through a single coordinating center, using a computer-generated block randomization in blocks of six.

### Setting

IB Salut is a public health organization in the Spanish National Health System, in which universal tax-funded health care services are provided to every citizen, and services are free at the point of access. Each patient is registered with one primary health care center. The Balearic Health Care Service covers the entire population of the Balearic Islands. Majorca is the largest island (850,000 inhabitants), and has four health districts (North, South, East and West Health Districts), each of which has a general hospital and several primary health care centers. PC services in Mallorca Island are provided by 3 PC units in non-acute hospitals and a PC home-based service, with 7 teams of physicians, nurses, and psychologists.

Primary health care centers from 3 health districts in Majorca will participate in the study. The intervention will be managed in the North Health District, which has 6 primary health care centers.

The intervention will be evaluated in 2 health districts: the South Health District (264,757 inhabitants), which has a university hospital and 14 primary health care centers, and the East Health District (326,988 inhabitants), which has a general hospital and 16 primary health care centers.

### Inclusion and exclusion criteria

Inclusion criteria: Primary health care centers from 2 health districts in Majorca.

Exclusion criteria: Unwillingness to participate.

### Intervention

The intervention consists of:

#### Appointment of palliative care leaders (nurses or doctors)

The leader will attend a 42 h training course in PC that is based on the “White Book” of PC training [29]. The role of the leader is to train and promote PC, and to act as a consultant and liaison between the PC home-based services and professionals in the primary health care services.

There will be regular contact with the leaders to monitor the implementation of the proposed activities, and to motivate and promote commitment towards the project. At the same time, leaders will be empowered by emphasizing the value of their roles at each meeting, their importance as “agents of change”, and the need for their commitment to assure the project’s success. Moreover, there will be biannual meetings, convened by the managing center of the Palliative Care Program of the Balearic Islands. The leaders in primary care, the PC home-care team, and the management of the primary health care team will attend these meetings. These meetings will review and discuss the leaders’ functions, assess and elaborate upon support documents, and identify the leaders’ training needs. A virtual shared space will be created using SharePoint (Microsoft) on the IB Salut corporate intranet to enable sharing of support documents, consultation papers, and all new information of interest to the leaders.

#### Identification of patients needing palliative care and classification according to case complexity

Leaders in the early identification and assessment of case complexity of patients receiving referral to PC home-based services will train primary health care doctors. Professionals will use two tools in the electronic clinical record: NECPAL CCOMS-ICO© [7] and e IDC-PAL [30].

**Fig. 2:**
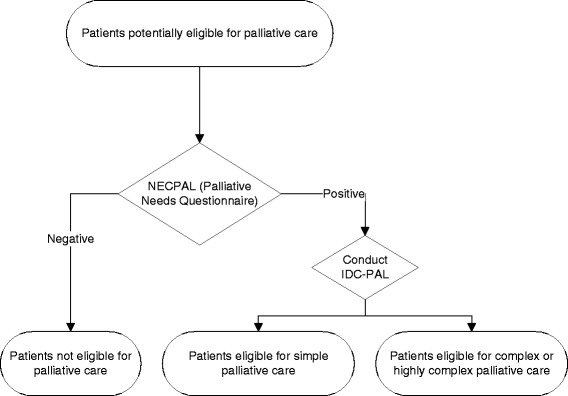
Identification and classification of patients needing palliative care

The NECPAL CCOMS-ICO© questionnaire was developed by the QUALY/CCOMS - ICO Observatory. It is based on the GSF-PIG [31] and SPICT [32], and adapted for the Spanish language. This questionnaire identifies patients in need of palliative measures, especially non-specific PC services (primary health care and general hospitalization). It is a screening tool (not a prognostic tool) for the early identification of patients needing PC. When a patient needing PC is identified, he/she is classified according to the complexity of the case to determine the most suitable, effective, and efficient care. IDC-PAL is a tool for determining the complexity of PC that is needed for patients with advanced-stage or terminal diseases [30]. This questionnaire considers situations and items that are identified after assessment of the patient-family unit by a multi-professional team. Based on the results of both questionnaires, patients will be classified as not eligible for PC, eligible for simple PC, or eligible for complex or highly complex PC (Fig. 2).

We have already developed training materials on the early identification of patients needing PC, and this includes a video about case complexity and the classification tools (IDC-PAL and NEC-PAL) and PowerPoint presentations to help explain the roles of primary health care leaders, GPs and nurses in PC.

#### Evaluation and integral management of needs

Professionals in primary health care teams have always cared for patients needing PC. We intend to give these professionals more skills and support resources, such as updated information, reference leaders in home-based PC services, diagnostic tools, and regular meetings for discussion of clinical cases with PC experts. Our main objective is to improve the care of non-hospitalized patients who require PC.

### Outcome measures

#### Primary outcomes

- Early identification (90 days before death), by GPs and nurses, of patients who are least 18 years-old and need PC based on electronic records.

#### Secondary outcomes

- Evaluation of case complexity among patients needing PC, based on electronic records.
- Patients who die at home.
- Total number of hospital admissions during the final month of life.
- Total number of emergency room admissions during the final month of life.
- Percentage of patients with any of the following “determinants of aggressive end-of-life care” during the final month of life [33]:
  - 1 or more admission to the emergency room.
  - 1 or more admission to the hospital.
  - 1 or more admission to the intensive care units (ICU).
  - More than 14 days in the hospital
- Cost of resources during the final month of life includes emergency department visits, outpatient office visits, primary health care visits, inpatient hospital stays, and ICU admissions. Total costs will be calculated by standard cost-per-unit prices, obtained from the Balearic official regional bulletin (17).

### Data collection

Early identification of patients, resource utilization, and place of death will be estimated relative to the number of patients who could benefit from PC services (causes of death: ICD codes C00-C97; 100–152, 160–169, N17, N18, N28, 112, 113, K70-K77, J06-J18, J20-J22, J40-J47, J96, G10, G20, G35; G122, G903, G231.F01, F03, G30, R54, B20-B24), using the calculation of the potential population size for PC developed by Rosenwax [8, 9]. The causes of death will be identified by review of the death certificates of patients who died 9 to 21 months after the onset of PC.

The NECPAL and IDC-PAL are integrated in the electronic records of GPs, thereby allowing early identification of patients needing PC and evaluation of case complexity. We will extract data from the electronic records upon completion of the NECPAL to identify the need for PC, estimate case complexity using IDC-PAL, and record all emergency department visits, outpatient office visits, primary care visits, duration of all hospital stays, and ICU admissions.

### Sample size

It is necessary to prospectively analyze 4640 clinical records of deceased patients from the defined population (causes of death: ICD codes C00-C97; 100–152, 160–169, N17, N18, N28, 112, 113, K70-K77, J06-J18, J20-J22, J40-J47, J96, G10, G20, G35; G122, G903, G231.F01, F03, G30, R54, B20-B24) to detect an increase of at least 5% in the proportion of patients identified as needing PC at 1 month before death in the intervention arm, assuming that 12% of patients will need PC in the control arm, a median of 263 patients in each health care center (cluster size), and an intra-class correlation coefficient of 0.015 [34].

### Statistical analysis

The effectiveness of the intervention will be evaluated by a prospective analysis of early patient identification for PC and resource utilization. Categorical baseline variables, at the patient level, from centers in the control and intervention groups will be compared using a 2-sided cluster-adjusted chi-squared test. The total number of admissions to a hospital or emergency room, visits to primary care, and total costs will be compared using Somers’ D rank correlation coefficient.

The estimated relative risk (RR) of being identified after the primary care intervention will be adjusted for cluster by a log link in a binomial distribution of a robust generalized estimating equation (GEE), with an exchangeable correlation structure. The absolute risk reduction (ARR) and number needed to treat (NNT) will be calculated from the RR.

We will conduct a multivariable regression analysis using a GEE, and determine a gamma distribution and log link to estimate the effectiveness of the intervention on health care costs.

A subgroup analysis will be used to determine the effectiveness of the intervention on the percentage of non-cancer patients identified as needing PC based on evaluation of case complexity. This procedure will analyze 3 different illness trajectories (cancer, organ failure, or dementia/frailty). The differential effectiveness of the intervention in these patients will be identified as a statistically significant interaction of the proposed variables with treatment efficacy.

## Discussion

Our objective of establishing PC leaders is to improve the PC provided by general and specific services. A more comprehensive and community-oriented approach to PC will provide better results if multiple health care professionals work together at the end of a patient’s life.

The role of primary health care is important for early identification of patients eligible for PC. The major aim of the proposed intervention is to increase the early identification of patients through the appointment of a specially trained PC leader (primary health care doctor or nurse) to each team. Thus, we expect that this intervention will sensitize doctors and nurses in the primary health care team so they can identify patients earlier using NECPAL, assess case complexity using IDC-PAL, and then provide essential care to patients requiring simple PC or more complex PC (home-based PC teams, or PC units in the hospital) when patient needs exceed their abilities, thereby avoiding hospitals admissions when possible.

The intervention efficacy will be measured as the increased early detection of patients needing PC, and by improvements in care and use of resources. To determine the effectiveness of the intervention, we will use secondary data from patients’ clinical records (administrative data), in each cases whose underlying cause of death was consistent with receipt of PC.

Previous studies have used secondary data from clinical records to assess health cost reductions in PC programs [35, 36] and to detect determinants of aggressive end-of-life care [33]. Aggressive or excessive care at the end-of-life can modify the course of disease, but at the expense of good management of symptoms and establishment of an advanced care plan [37]. We are aware of the limitations of assessing the quality of PC based on “service use” and “care management”, rather than factors more directly related to the patient’s and family’s biological, social, and psychological experiences [38]. Many studies have examined the use of aggressive care at the end of life [33, 39, 40]. There is general agreement that a patient spending many hours of the last month of life in an acute care bed or repeatedly accessing acute care emergency services are indicators of poor care at the end of life [37, 38, 41]. Information in patients’ electronic records and variables taken from deceased patients’ records have great value for research and training. Electronic health care records enable researchers to follow patients more easily [42], and to assess the efficacy of programs that seek to change the management system. The strategy of measuring the effect of an intervention via database review is not yet common in RCTs, but it avoids the recruitment problem, the response shift, ethical problems, and selection bias [43]. We will analyze all randomized centers, even if they do not consolidate the role of PC leader or implement the proposed intervention, in our intention to treat analysis.

Another limitation of this study is the method used to determine which patients should receive PC [9, 24]. It is not possible to identify all patients who have a condition making them potentially eligible for PC. We estimated that approximately 75% of patients who die due to cancer, organ failure, and dementia/fragility could be helped by early identification for PC. Moreover, because we cannot identify all potentially eligible patients, if our proposed intervention shows efficacy under the conditions of this study, this indicates that its actual effect could be even greater.

If our results show a positive effect of the intervention, this approach could be replicated in other countries where primary health care is organized into teams, and the GP acts as the gatekeeper.

## Conclusion

The proposed intervention introduces PC leaders to health care centers who have the opportunity to share their experiences with the health care team and access documents to prepare for team training sessions Thus, the PC leader is a part of the PC scientific and professional community, and is recognized by the health care organization. Therefore, the proposed intervention is multidimensional, formative, and organizational. A multidimensional approach is the most effective method to achieve changes in clinical practice for end-of-life care [44, 45]. Finally, we are sure that the project will benefit all professionals in primary health care, because all the training and support materials generated will be accessible through the primary health care intranet and the web. We will also use the project as a test system for obtaining clinical indicators from electronic medical records, and this will help with assessment of the PC program in future developments.

The appointment of PC leaders to primary health care teams could improve the early detection of patients who are eligible for PC, increase the use of the complexity assessment tool (IDC-PAL) for patients entering PC, improve the application of essential PC to cases requiring simple PC and specialized services when needed for more complex cases. All this implies a more rational use of resources. Trained primary health care professionals [46] [22], and with the advice of PC leaders when needed, can help to improve PC when patients are at home.

## Abbreviations

ARR: Absolute risk reduction
EAPC: European atlas of palliative care
GEE: Generalized estimating equation
GPs: General practitioners
ICU: Intensive care units
NNT: Number needed to treat
PC: Palliative care
PHCC: Primary Health Care Centers
RCT: Randomized clinical trial
WHO: World Health Organization
